# FHA-Mediated Cell-Substrate and Cell-Cell Adhesions Are Critical for *Bordetella pertussis* Biofilm Formation on Abiotic Surfaces and in the Mouse Nose and the Trachea

**DOI:** 10.1371/journal.pone.0028811

**Published:** 2011-12-22

**Authors:** Diego O. Serra, Matt S. Conover, Laura Arnal, Gina Parise Sloan, María E. Rodriguez, Osvaldo M. Yantorno, Rajendar Deora

**Affiliations:** 1 Facultad de Ciencias Exactas, Centro de Investigación y Desarrollo en Fermentaciones Industriales (CINDEFI), CONICET-CCT-La Plata, Universidad Nacional de La Plata, La Plata, Argentina; 2 Program in Molecular Genetics, Wake Forest University Health Sciences, Winston-Salem, North Carolina, United States of America; 3 Department of Microbiology and Immunology, Wake Forest University Health Sciences, Winston-Salem, North Carolina, United States of America; East Carolina University School of Medicine, United States of America

## Abstract

*Bordetella* spp. form biofilms in the mouse nasopharynx, thereby providing a potential mechanism for establishing chronic infections in humans and animals. Filamentous hemagglutinin (FHA) is a major virulence factor of *B. pertussis*, the causative agent of the highly transmissible and infectious disease, pertussis. In this study, we dissected the role of FHA in the distinct biofilm developmental stages of *B. pertussis* on abiotic substrates and in the respiratory tract by employing a murine model of respiratory biofilms. Our results show that the lack of FHA reduced attachment and decreased accumulation of biofilm biomass on artificial surfaces. FHA contributes to biofilm development by promoting the formation of microcolonies. Absence of FHA from *B. pertussis* or antibody-mediated blockade of surface-associated FHA impaired the attachment of bacteria to the biofilm community. Exogenous addition of FHA resulted in a dose-dependent inhibitory effect on bacterial association with the biofilms. Furthermore, we show that FHA is important for the structural integrity of biofilms formed on the mouse nose and trachea. Together, these results strongly support the hypothesis that FHA promotes the formation and maintenance of biofilms by mediating cell-substrate and inter-bacterial adhesions. These discoveries highlight FHA as a key factor in establishing structured biofilm communities in the respiratory tract.

## Introduction

*Bordetella pertussis* is the major causative agent of whooping cough (pertussis), a highly infectious and transmissible disease of the human upper respiratory tract. Over the past two decades pertussis has experienced a resurgence worldwide [1]. While pertussis is traditionally described as a severe childhood disease, it is also common among adolescents and adults, in whom it often manifests as persistent cough with milder symptoms [2], [3]. A large body of evidence strongly supports that adolescents and adults as reservoirs and sources of pertussis transmission [4], [5], [6], [7]. In a recent population based study conducted over a period of about two years in the Netherlands, 53% of the household contacts had laboratory-confirmed pertussis. Strikingly, in 60% of the households, the source of transmission to infants was clearly established to be one of the family members [8].

We recently began to explore the capacity of *B. pertussis* to form biofilms, postulating that this pathogen adopts the biofilm lifestyle as a strategy to survive in the human respiratory tract [9], [10], [11], [12]. A biofilm is a complex community-based mode of existence that microbes establish over abiotic or living surfaces [13]. This lifestyle confers upon pathogens several advantageous traits linked to virulence, including resistance to environmental stress, host defences and antimicrobial compounds [14], [15]. Such virulence-related traits ultimately facilitate the establishment of persistent infections or a carrier state [16]. The existence of a biofilm mode of life for *B. pertussis* and its closely related species *B. bronchiseptica* in the mouse respiratory tract has been demonstrated [9], [10], [17]. One of the strengths of this model is that the *Bordetella* cells attached to the respiratory epithelium are surrounded by a self-produced extracellular matrix composed of the Bps polysaccharide, thereby satisfying the definition of *in vivo* biofilms. Moreover, *B. pertussis* has been found adherent to ciliated cells of explant cultures and in tissue biopsies of pertussis patients in the form of clusters and tangles (structures reminiscent of biofilms) [18], [19], [20]. Taken together, while these results suggest an important role for the *B. pertussis* biofilm state in human infections, the knowledge of the events and factors involved in this lifestyle is rudimentary.

Biofilm development depends on the ability of microorganisms to establish cell-substrate and cell-cell interactions, since they support sessile growth and the development of multicellular structures [21]. To promote these interactions, most biofilm-forming bacteria make use of surface-associated structures such as fimbriae, Type IV pili, outer membrane proteins or exopolymers like polysaccharides [22], [23], [24], [25], [26], [27], [28]. *B. pertussis* produces various factors implicated in bacterial attachment, which promote sequential and/or redundant interactions with host epithelia in the respiratory tract. Among such factors are adhesins like filamentous hemagglutinin (FHA), pertactin and fimbriae [29]. FHA is a large, β-helical, highly immunogenic protein that is both surface associated and secreted [30]. FHA displays diverse attachment activities derived from its multiple binding domains, which facilitate *Bordetella* attachment to a variety of eukaryotic cell types and extracellular structures in the respiratory epithelium [31]. Other documented roles include mediating *B. pertussis* invasion to host cells, triggering immunomodulatory responses and promoting autoagglutination and colonization of the respiratory tract [32], [33], [34], [35], [36], [37], [38]. FHA is the prototypical member of the family of bacterial proteins secreted by the two-partner secretion system [39]. Two members of this family, CdrA in *P. aeruginosa* and XacFhaB in *Xanthomonas axonopodis* pv. Citri have recently been shown to be involved in biofilm formation [40], [41].

Thus, although the existing literature justifies consideration of FHA as a candidate for biofilm adhesin, there is little known on its potential role in biofilm formation. It has been shown that FHA contributes to biofilm formation by the animal pathogen *B. bronchiseptica* [36], [42]. In the case of the human pathogen *B. pertussis*, the role of this protein factor has remained unexplored. Moreover, the mechanism by which FHA contributes to biofilm formation in either *B. bronchiseptica* or *B. pertussis* is unknown.

In the present study, we investigated the mechanism by which *B. pertussis* FHA mediates the different steps of biofilm development on abiotic surfaces. We show that FHA is a key factor for bacterial attachment and subsequent cell accumulation on substrates. Our results indicated that surface-associated FHA promotes the formation of structured biofilms by mediating inter-bacterial adhesion. We extended our *in vitro* observations by investigating the function of FHA in biofilm formation in the mouse respiratory tract. Absence of FHA caused a significant reduction in the ability of *B. pertussis* to form and maintain biofilm-like structures in the mouse nose and the trachea. Together our results highlight the role of FHA as a central structural factor in *B. pertussis* biofilm development. We propose that FHA-mediated biofilm formation in the respiratory tract allows *B. pertussis* to evade the host immune response thereby establishing a bacterial reservoir in the human respiratory tract.

## Results

### *B. pertussis* FHA is required for biofilm formation on abiotic surfaces

**Figure 1:**
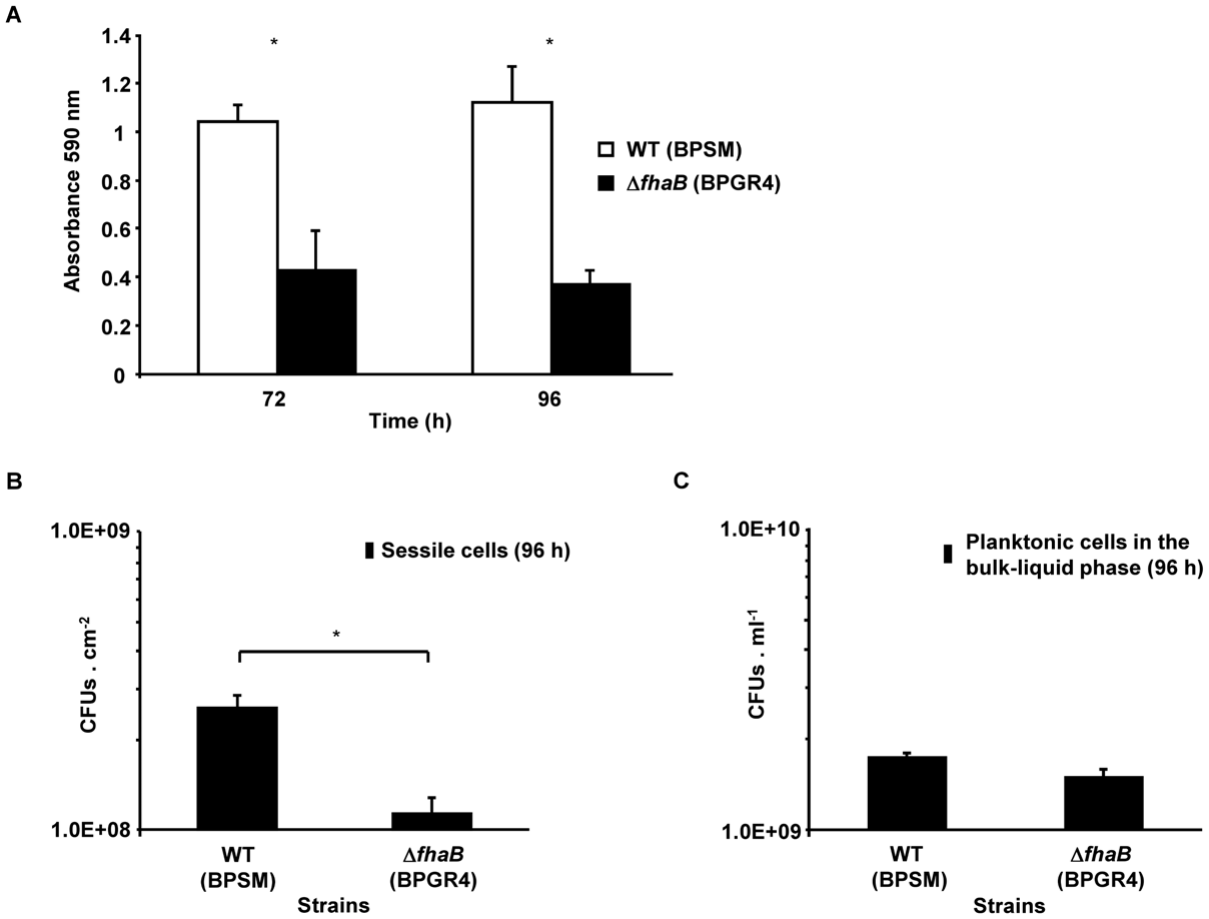
Biofilm formation by WT (BPSM) and Δ*fhaB* (BPGR4) *B. pertussis* strains on abiotic surfaces. Biofilm cultures were performed in glass column systems containing polypropylene beads. (A) Biofilm biomass accumulated by each strain over the polypropylene beads after 72 and 96 h of cultivation was stained with CV 0.1% (v v^−1^). The CV stain associated with cells was solubilized in ethanol/acetone (80∶20) and the resulting solution was subjected to measurement of the absorbance at 590 nm. The data are the means ± standard deviations of three independent experiments. An asterisk indicates significant differences between the WT and the Δ*fhaB* mutant (*P* value <0.05; Student's *t*-test). (B) Viable cell counts of the biofilm cells of each strain. The adhered cells were gently washed and detached from the polypropylene beads by slight agitation in PBS buffer. Serial dilutions of cell suspensions were then plated on BGA plates. Results are expressed as the number of colony-forming units per unit area (CFUs cm^−2^). The data are the means ± standard deviations of three independent experiments. An asterisk indicates significant differences between the WT and the Δ*fhaB* mutant (*P* value <0.05; Student's *t*-test). (C) Viable cell counts of the planktonic cells in the bulk-liquid phase of biofilm cultures of each strain. The cells were drained out of the biofilm column system at the end of the experiment (96 h). Serial dilutions of cell suspensions were then plated on BGA plates. Results are expressed as the number of colony-forming units per unit volume (CFUs ml^−1^). The data are the means ± standard deviations of three independent experiments.

No information is available on the role of FHA in contributing to the biofilm development of *B. pertussis*. We initiated this study by utilizing a previously described glass column culture system packed with polypropylene beads (the growth support) [11] to examine biofilm formation under aeration conditions. The wild-type (WT) strain BPSM, a Tohama I derivative, and the isogenic Δ*fhaB* mutant, BPGR4, which carries a chromosomal deletion of the FHA structural gene *fhaB* [43], were similarly cultured in this system for 72 and 96 h. We have previously shown that within this time frame of cultivation, *B. pertussis* biofilms gain structural maturity [11], [44]. The biofilm biomass for the two strains was quantified by crystal violet staining and measuring the absorbance of the solubilized stain at 590 nm. Compared to the WT strain, the Δ*fhaB* mutant was defective in its ability to form biofilms after 72 h of growth. Even after 4 days, the mutant strain displayed significantly reduced capacity to accumulate biofilm biomass (Fig. 1A). Similar results were obtained when borosilicate beads were used as growth support (data not shown). We also confirmed the crystal violet staining results by determining the number of viable sessile cells of each strain recovered from the polypropylene beads at the end of the experiment (96 h). The number of CFUs obtained for the mutant strain was significantly lower than that obtained for the WT strain (P<0.05, *t* test) (Fig. 1B). The observed differences in surface-attachment between the WT and the Δ*fhaB* strains were not due to their differential ability to grow either planktonically or in shaking cultures. The number of Δ*fhaB* cells present in the bulk-liquid phase at 96 h was not significantly different from that of the WT strain (*P* = 0.11, *t* test) (Fig. 1C). Moreover, these two strains exhibited similar specific growth rates in shaking cultures (data not shown). These results suggest that FHA contributes to the formation of biofilms by *B. pertussis*.

### The biofilm-forming defect of the ΔfhaB mutant derives from its impaired capacity to interact with the substrate during attachment, and subsequent stages of development

**Figure 2:**
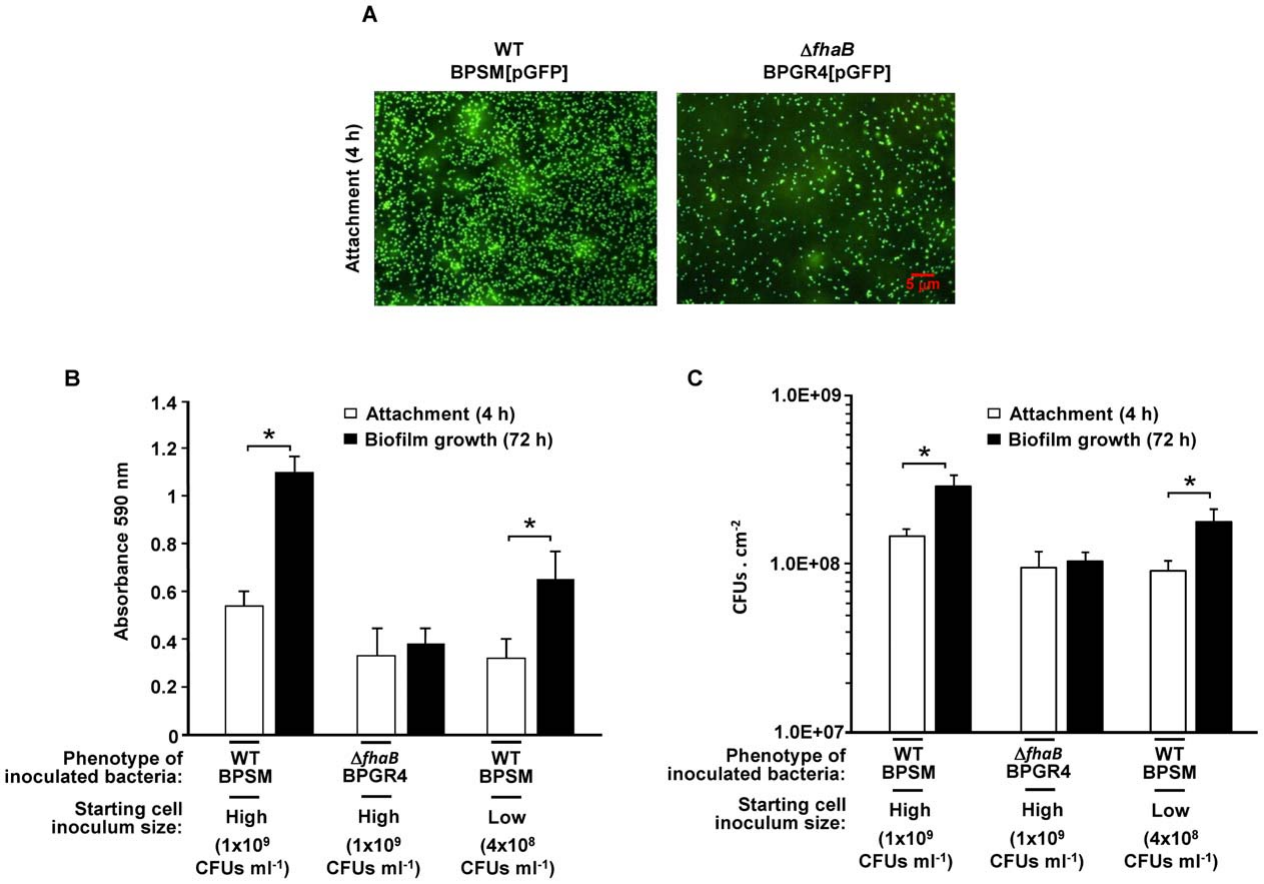
Early attachment of *B. pertussis* strains to abiotic surfaces. (A) Attachment of WT and Δ*fhaB* strains to glass. Inoculated bacteria (1×10^9^ CFUs ml^−1^) were allowed to attach to the surface under static conditions for 4 h at 37°C. Fluorescence images of WT and Δ*fhaB* GFP-tagged cells that remained adhered to the glass coverslips. (B) Attachment and subsequent biofilm growth by the WT and Δ*fhaB B. pertussis* strains. WT and Δ*fhaB* biofilm cultures were initiated with high inoculum levels (1×10^9^ CFUs ml^−1^). Bacteria were allowed to attach for 4 h followed by incubation under normal biofilm culture conditions. The effect of low-level attachment on subsequent biofilm formation by the WT *B. pertussis* strain was also examined. The number of WT bacteria in the inoculum was reduced from 1×10^9^ to 4×10^8^ CFUs ml^−1^ so as to decrease the population of cells initially adhered to the level displayed by the Δ*fhaB* strain with a high inoculum (1×10^9^ CFUs ml^−1^), and then incubated under normal biofilm culture conditions. Adhered biomass was stained with CV 0.1% (v v^−1^). The CV stain associated with cells was solubilized in ethanol/acetone (80∶20) and the resulting solution was subjected to measurement of the absorbance at 590 nm. The data are the means ± standard deviations of three independent experiments. An asterisk indicates significant differences between attachment and biofilm growth (*P* value <0.05; Student's *t*-test). (C) Viable cell counts of the adhered cells of each strain to polypropylene beads under the conditions described in B. The adhered cells were gently washed and detached from the polypropylene beads by slight agitation in PBS buffer. Serial dilutions of cell suspensions were then plated on BGA plates. Results are expressed as the number of colony-forming units per unit area (CFUs cm^−2^). The data are the means ± standard deviations of three independent experiments. An asterisk indicates significant differences between attachment and biofilm growth (*P* value <0.05; Student's *t*-test).

Biofilm development in bacteria is a multi-stage process and results from initial surface attachment of bacterial cells followed by accumulation into multilayered cell clusters. We therefore asked whether the observed biofilm defect of the Δ*fhaB* mutant resulted from defects in initial attachment. To investigate this, we first compared the attachment of GFP-tagged derivatives of both the WT and the Δ*fhaB* strains to glass coverslips by fluorescence microscopy. In order to allow the bacteria present in the liquid phase to reach the substrate, a 4-h incubation period was established for assaying attachment. Note that this time period is shorter than the doubling time of both the *B. pertussis* strains (data not shown) and thus the attachment is considered independent from growth. Images in Fig. 2A show that after 4 h of incubation, a greater number of GFP-tagged cells of the WT strain remained attached to the surface relative to that for similarly tagged Δ*fhaB* mutant. In parallel, we also examined the attachment of the WT and the Δ*fhaB* strains to polypropylene beads. The adhered cell biomass was quantified by crystal violet staining. Results show that after 4 h of incubation, the Δ*fhaB* mutant exhibited a significantly reduced percentage of cell biomass adhering to polypropylene beads compared to that of the WT strain (Fig. 2B, compare first and second white bars).

While these results indicate that FHA is necessary for efficient initial attachment of *B. pertussis* to the surface, they do not provide information on the extent to which the observed attachment defect, resulting from the absence of FHA, affects subsequent steps of biofilm formation. Thus, we next investigated whether a low number of adherent bacteria, similar to that found for the Δ*fhaB* mutant strain (Fig. 2B, second white bar), was sufficient to result in a late-stage biofilm. To this end, the number of WT bacteria in the inoculum was reduced from 1×10^9^ to 4×10^8^ CFUs ml^−1^. Inoculated bacteria were allowed to attach for 4 h followed by incubation under normal biofilm culture conditions. Under these conditions, while the WT strain was capable of forming biofilms (Fig. 2B, third black bar), the Δ*fhaB* mutant did not show an increase in the biofilm biomass (Fig. 2B, second black bar). We also confirmed the crystal violet staining results by determining the number of viable cells of each strain recovered from the polypropylene beads in the same experiment (Fig. 2C). Together these results suggest that the absence of FHA renders *B. pertussis* not only deficient in early attachment, but also in the accumulation of the biofilm biomass.

### The reduced ability of the ΔfhaB mutant to form biofilms correlates with defects in the formation of structured cell clusters

**Figure 3:**
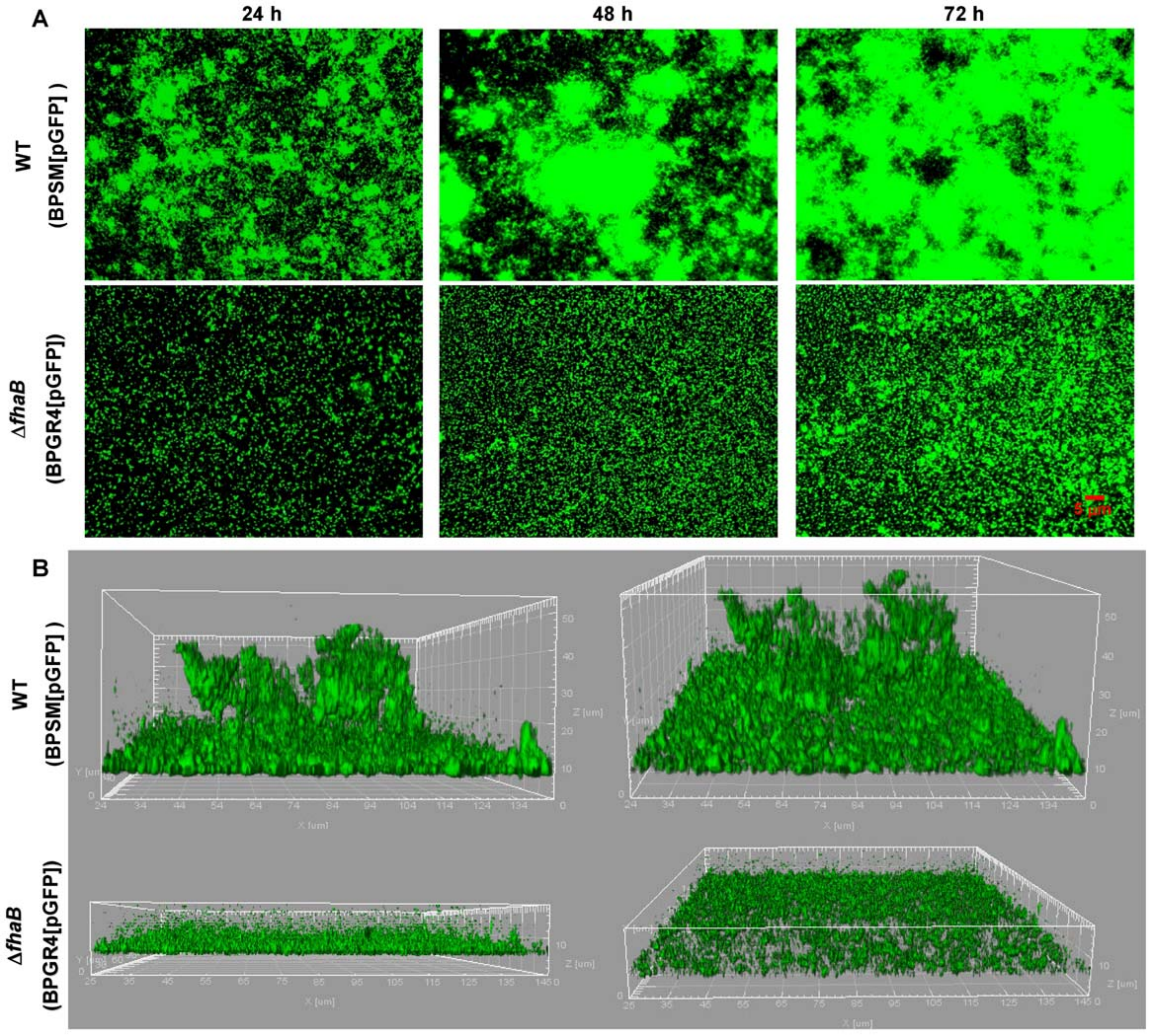
Microscopy analysis of biofilm formation by the WT and the Δ*fhaB* strains. GFP-tagged strains were inoculated directly in the continuous-flow chambers. Bacteria were allowed to attach for 4 h at 37°C, and then the sterile SS medium was pumped through the flow chambers at a flow rate of 0.1 ml min^−1^. The experiment was repeated at least three times. Biofilms were visualized *in situ* by fluorescence and CLSM microscopy. (A) Representative fluorescence micrographs of biofilms taken every 24 h for 3 days at a magnification of ×400. (B) Volumetric 3D reconstructions of representative Z-section image stacks of biofilms taken at 72 h of growth at a magnification of 630×. For each strain, images are presented in two different perspectives.

The absence of FHA strongly diminished, but did not fully abolish biofilm accumulation. To better understand the function of FHA in biofilm development, structural aspects of this mode of growth were examined. GFP-tagged WT and mutant strains were cultured in continuous-flow chamber systems. These systems offer dynamic flow conditions, which allow the continuous replenishment of nutrients, as well as analytical advantages like non-invasive real-time monitoring of structural development of biofilms by microscopy techniques. At indicated time points, biofilm formation of each strain was examined *in situ* by fluorescence microscopy. As has been previously shown [10], [12], by 24 h, the WT strain was adherent to the glass surface in a diffused manner and formed small clusters of cells (Fig. 3A). Between 48 and 72 h of cultivation, the density of surface attached WT cells increased and at this stage biofilms appeared structurally complex. Cells were observed to be arranged in large and irregularly shaped microcolonies attached to the surface. In contrast, a reduced number of Δ*fhaB* mutant cells were attached to the coverslips at 24 h with no visible cell clusters. With time, while the number of Δ*fhaB* mutant cells attached to the borosilicate surface slightly increased, structures resembling those of the WT biofilm were never observed. The increase in biomass for the mutant strain mainly correlated with the presence of single cells dispersed over the surface or with the formation of diffuse small cell clusters. These data suggest that the Δ*fhaB* mutant is not only delayed in biofilm formation but is unable to form a typical structured biofilm community.

To gain information on the differences in the structural features of the biofilms formed by the WT and the mutant strains, 72-h-old biofilms were examined by Confocal Laser Scanning Microscope (CLSM). Stacks of Z-section images were collected and reconstructed into 3D images. As observed in Fig. 3B, after 72 h of growth, the WT strain formed robust biofilms consisting of large tower-shaped cell aggregates. In contrast, the mutant strain developed minute clusters across the whole substrate. These image stacks were further analyzed by the COMSTAT software, which allowed us to extract quantitative information of biofilm architecture [45]. The descriptors chosen were: mean and maximum thickness, total biomass, and the mean size of cell clusters (microcolonies). Biofilms developed by the WT strain exhibited a mean thickness of 25 µm and a maximum thickness of approximately 48 µm (Table 1). In contrast, the Δ*fhaB* strain formed a non-uniform layer of cells on the surface with a mean thickness of only 2.5 µm and maximum thickness of about 4 µm. Comparison of the biomass values also showed significant differences in the density of cells accumulated on the surface between the two strains. Similarly, computation of the average of the mean area of cell clusters located at the substratum showed that the Δ*fhaB* mutant formed only small cell clusters as compared to the WT strain (Table 1). In conclusion, COMSTAT analyses of CLSM image stacks of the biofilms suggest that FHA is critical for the development of the three-dimensional architecture of *B. pertussis* biofilms, as evidenced by the thick and voluminous biofilms produced by the WT strain compared to the relatively flat, dispersed and monolayer-like mat of cells produced by the Δ*fhaB* strain.

**Table 1:** Quantitative analysis of structural features of biofilms formed by *B. pertussis* WT and Δ*fhaB* strains after 72 h of cultivation^a^

Parameter^b^ Strain	WT (BPSM[pGFP])	Δ*fhaB* (BPGR4[pGFP])	*P* value^c^
Maximum thickness (µm)	47.6 (0.84)	3.82 (0.18)	1.1 10^−7^
Mean thickness (µm)	24.85 (1.65)	2.47 (0.36)	2.2 10^−5^
Biomass d (µm^3^.µm^−2^)	5.28 (0.53)	1.29 (0.20)	0.0003
Mean cell cluster area^e^ (µm^2^)	426.75 (95.9)	14.54 (2.88)	0.0016

a Biofilms were grown on the base of flow chambers under continuous flow conditions.

CLSM images stacks were acquired from random points by using a plan-neofluar ×100/1.3 oil immersion objective, at 1 µm z-intervals down through the biofilm. Image stacks were analyzed by COMSTAT.

b Average values of parameters obtained from COMSTAT analysis of six CLSM image stacks derived from three independent experiments. Standard errors are given in parentheses.

c *P* values were determined using an unpaired two-tailed Student's *t*-test.

d The value is biomass volume divided by substratum area.

e The function calculates the mean area size of cell clusters located at the substratum.

### The presence of FHA on the surface of *B. pertussis* cells is necessary to mediate the formation of biofilms

From the above results, it is clear that FHA is involved not only in the interaction of *B. pertussis* with surfaces, but also in the formation of multicellular cell clusters. We hypothesized that FHA may facilitate inter-bacterial adhesion, thereby resulting in the formation of microcolonies and eventual development of mature biofilms. To examine this hypothesis, we first investigated if the presence of this protein on the bacterial surface was needed to integrate bacteria into the biofilm community. We compared biofilms formed by mixtures of the WT and the Δ*fhaB* strains carrying different antibiotic resistance genes. The CFUs of each strain in the mixed biofilms were enumerated by plating on selective BGA plates. When WT (B213[pGFP], Gm^r^) and Δ*fhaB* (BPGR4, Nal^r^) strains were simultaneously co-cultured in biofilm, the population of cells recovered after 72 h was mainly composed of WT cells (91%). In contrast, in a similar control experiment, when the two WT strains with different antibiotic resistance (B213[pGFP] Gm^r^ and BPSM Nal^r^) were co-inoculated, the fractions of cells of each strain recovered from the mixed biofilm were not substantially different (Table 2). Competition growth experiments carried out in parallel as control showed that the growth rate of each strain was not affected by the presence of the other strain (data not shown). Moreover, both the WT strains, B213 and BPSM, which were derived from Tohama I, exhibited similar growth in shaking cultures (Fig. S1) and accumulate biofilm biomass at similar levels (Fig. S2). It has also been previously shown that the plasmid CW504, herein called pGFP, does not affect either growth or FHA expression of *Bordetella* [46], [47], [48]. Data presented in Fig. S3 further show that the presence of the pGFP plasmid does not affect the biofilm forming ability of *B. pertussis*.

**Table 2:** Quantification of bacteria recovered during simultaneous and sequential co-inoculation biofilm assays^a^.

Co-inoculated strains		Mode and time of inoculating strain B (Time; h)	Fraction of bacteria recovered at 72 h A/B
A	B		
WT (B213[pGFP], Gm^r^)	Δ*fhaB* (BPGR4; Nal^r^)	Simultaneous (0)	0.91/0.09
WT (B213[pGFP], Gm^r^)	WT (BPSM; Nal^r^)	Simultaneous (0)	0.56/0.44
WT (B213[pGFP], Gm^r^)	Δ*fhaB* (BPGR4; Nal^r^)	Sequential (24)	0.95/0.05
WT (B213[pGFP], Gm^r^)	WT (BPSM; Nal^r^)	Sequential (24)	0.63/0.37

a Simultaneous and sequential co-inoculation biofilm assays were performed in glass column systems packed with polypropylene beads, which constituted the growth support.

After 72 h of cultivation, the biofilm cells were detached from the beads and resuspended in PBS. Appropriate dilutions were plated on selective BGA plates containing Gm or Nal and enumerated. Population of cells of each strain (A or B) is expressed as a percentage of the total population of cells recovered.

We also examined whether the Δ*fhaB* strain could be integrated into an established biofilm formed by the WT strain. To test this, WT (B213[pGFP], Gm^r^) was cultured for 24 h on polypropylene beads as described below. After 24 h of incubation, equal numbers of either the Δ*fhaB* (BPGR4, Nal^r^) or the WT (BPSM, Nal^r^) cells were inoculated into the culture system and cultured for an additional 48 h. Results showed in Table 2 indicate that even when co-cultured sequentially, the Δ*fhaB* mutant was severely impaired in its capacity to be integrated into pre-existing biofilms. On the contrary, co-inoculated WT (BPSM, Nal^r^) cells were found to be incorporated into the biofilm initiated by another WT (B213[pGFP], Gm^r^) strain, although to a lesser extent compared to when they were inoculated simultaneously. Taken together, these results suggest that the presence of FHA on the cell surface is needed for the bacteria to become part of the biofilm community. These results also suggest that surface-associated FHA on the WT bacterial cell does not compensate for this protein on the surface of a FHA-deficient bacterium.

### Inhibition of the attachment of *B. pertussis* cells to the biofilm by anti-FHA serum

**Figure 4:**
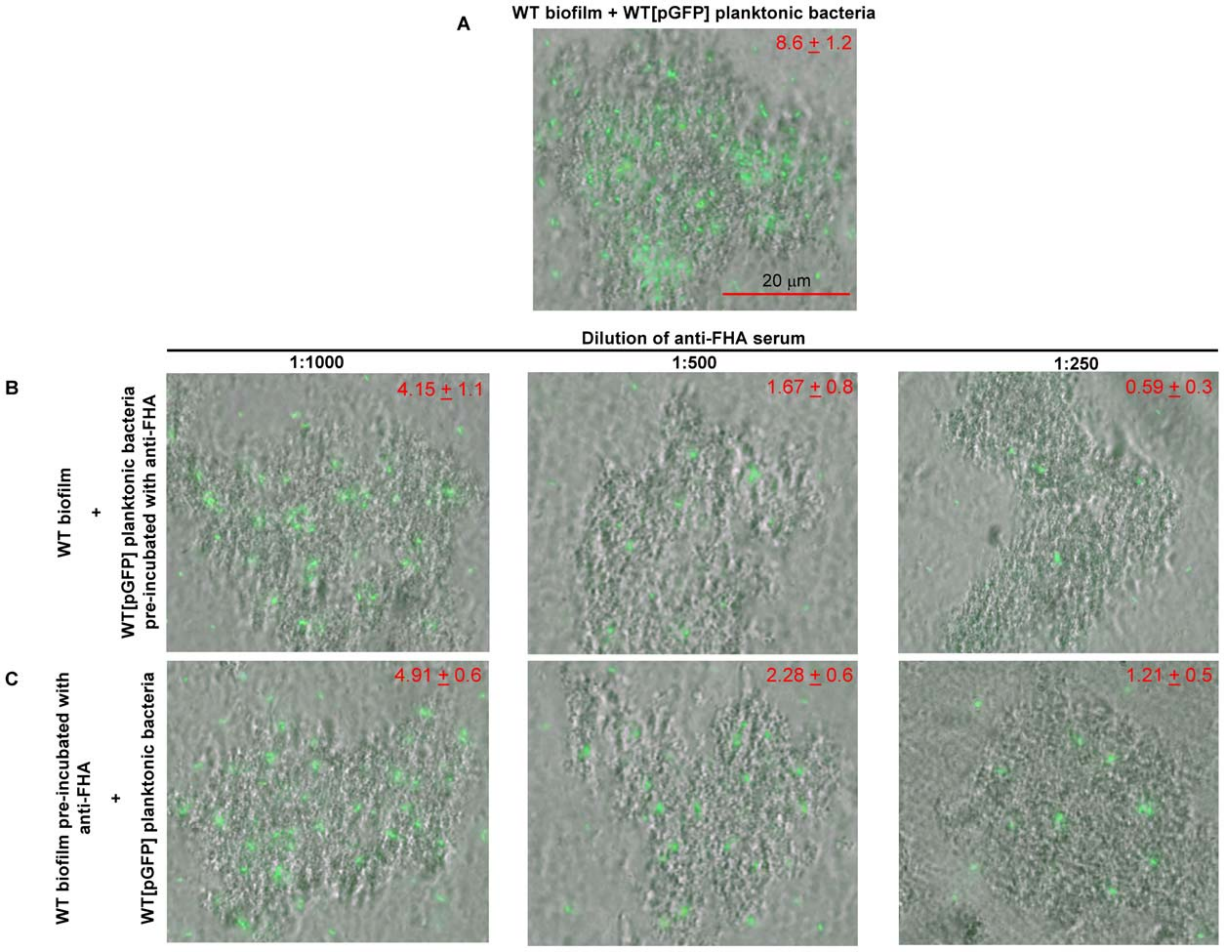
Inhibition of association of *B. pertussis* planktonic cells with the biofilms by anti-FHA serum. GFP-tagged planktonic bacteria of the WT (BPSM) strain, pre-incubated with SS medium (A) or with (B) dilutions of anti-FHA serum, followed by incubation for 4 h with 24-h-old WT biofilms. (C) Twenty four-h-old WT biofilms pre-incubated with dilutions of anti-FHA serum, and then subjected to a 4-h incubation period with planktonic GFP-tagged WT bacteria. GFP-expressing bacteria attached to the biofilms were visualized by acquiring multi-channel (transmitted light and fluorescence) CLSM images. At least six microscopic fields per coverslips and four coverslips per condition were analysed. A representative merged image for each condition is shown. The data indicated in the top right of each image are the means ± standard deviations of the numbers of GFP-tagged bacteria per 100 µm^2^ of pre-formed biofilm microcolony derived from the analysis of twenty four microscopic fields on four coverslips per condition. The numbers of attached bacteria was determined by examining multi-channel images using ImageJ in conjunction with the ITCN plug-in.

To further substantiate the role of surface-associated FHA in mediating bacterial association with the biofilms, we examined the effect of anti-FHA serum on the attachment of *Bordetella* cells to the biofilms. GFP-tagged planktonic bacteria of the WT strain were pre-incubated with different dilutions of the anti-FHA serum and allowed to attach to 24-h-old biofilm of the same strain. The attachment level was determined by visualizing and enumerating the GFP-expressing bacteria that colocalized with the biofilms. Untreated WT bacteria attached uniformly to the pre-existing biofilm microcolonies (Fig. 4A). On an average, 8.6±1.2 GFP-tagged WT cells were found to adhere per 100 µm^2^ of pre-formed WT biofilm microcolony. Pre-incubation of GFP-tagged WT bacteria with anti-FHA serum resulted in a decrease of bacterial attachment to the pre-formed biofilms in a dose-dependent fashion (Fig. 4B). Maximum decrease in adherence was obtained at the 1∶250 dilution of the serum, which resulted in an average of 0.59±0.3 attached cells/100 µm^2^ of WT biofilm microcolony.

Next, we determined if FHA present on the surface of the biofilm bacteria contributed to the attachment of planktonic bacteria to the biofilms. To test this, 24-h-old WT biofilms were pre-incubated with the anti-FHA serum, and then subjected to a 4-h incubation period with planktonic GFP-tagged WT bacteria (Fig. 4C). At the 1∶250 dilution of the anti-FHA serum, only 1.21±0.5 GFP-tagged WT cells/100 µm^2^ of WT biofilm microcolony were found to be attached. Control experiments where either the planktonic or the biofilm bacteria were pre-incubated with normal sheep serum did not interfere with the adherence of planktonic WT bacteria to the biofilms (Fig. S4). Moreover, pre-incubation of Δ*fhaB* planktonic cells with the anti-FHA serum did not further reduce the observed low level of attachment of the Δ*fhaB* mutant (data not shown). In combination, these results argue that the presence of FHA on the surface of both the planktonic and the biofilm bacteria is required for promoting the association of the bacterial cells with the *Bordetella* biofilms.

### Addition of exogenous FHA prevents association with the biofilms

**Figure 5:**
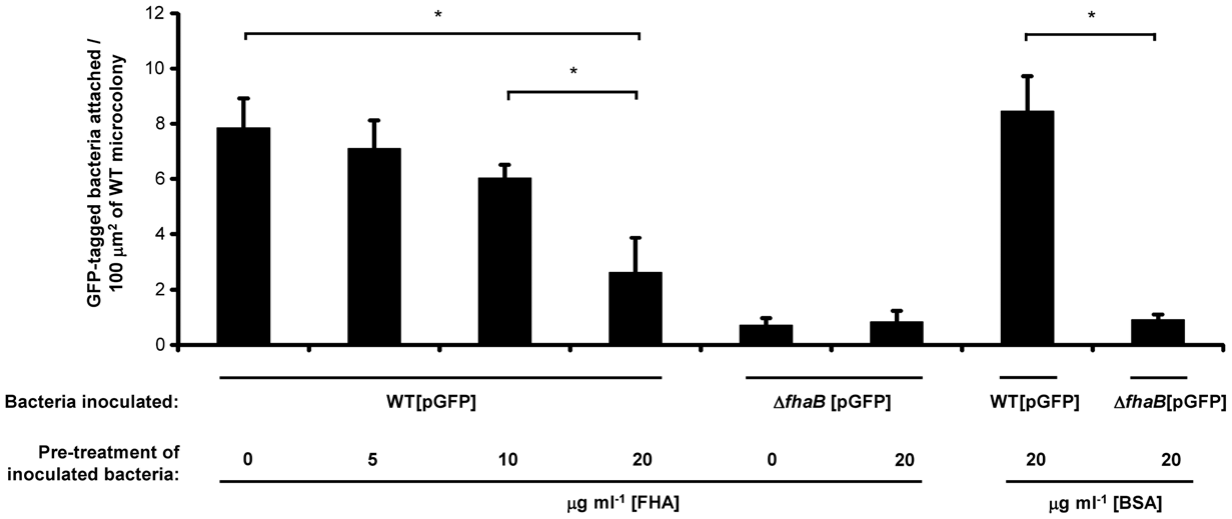
Association to biofilms of *B. pertussis* planktonic cells pre-treated with exogenous FHA. GFP-tagged planktonic bacteria of the WT (BPSM) and Δ*fhaB* (BPGR4) strain pre-incubated with SS medium, various concentrations of purified FHA or BSA (20 µg ml^−1^) followed by incubation for 4 h with 24-h-old WT biofilms. GFP-expressing bacteria attached to biofilms were visualized by acquiring multi-channel (transmitted light and fluorescence) CLSM images. The numbers attached bacteria was determined by examining multi-channel images using ImageJ in conjunction with the ITCN plug-in. The data are the means ± standard deviations of the numbers of GFP-tagged bacteria per 100 µm^2^ of preformed biofilm microcolony derived from the analysis of twenty four microscopic fields on four coverslips per condition. An asterisk indicates significant differences between the conditions compared (*P* value <0.05; Student's *t*-test).

Based on the above results, we hypothesized that externally added FHA will bind to surface-associated FHA and block inter-bacterial adhesion. To test this, we evaluated the extent to which GFP-tagged planktonic bacteria of the WT strain, pre-incubated with different concentrations of purified FHA, attach to 24-h-old biofilms of the same strain. As indicated above, the attachment level was determined by visualizing and enumerating the GFP-expressing bacteria that colocalize with the biofilms. We found that the attachment of planktonic WT bacteria to the biofilms decreased in response to increasing concentration of FHA (Fig. 5). Under the assay conditions, maximum effect of FHA in reducing the attachment of WT bacteria was observed at a concentration of 20 µg ml^−1^. In parallel, control experiments were conducted with either the Δ*fhaB* strain or with BSA. Compared to the WT strain, the Δ*fhaB* mutant exhibited a low level of attachment, which could not be further reduced by pre-incubation with 20 µg ml^−1^ FHA (Fig. 5). Likewise, pre-incubation of planktonic bacteria of the WT strain or the Δ*fhaB* strain with 20 µg ml^−1^ BSA resulted in attachment levels that did not differ significantly from those exhibited by each strain without pre-exposure to proteins.

In total, by utilizing a combination of multiple experimental approaches, we have obtained strong evidence that FHA is necessary for biofilm formation of *B. pertussis* on abiotic surfaces.

### FHA promotes formation of biofilms in the respiratory tract

The intranasal mouse model of *Bordetella* infection is widely utilized for pathogenic studies and has recently been demonstrated by us to be a valid and powerful prototype for examining bacterial biofilm development *in vivo* [9], [10]. Utilizing this model, we have shown that both *B. pertussis* and *B. bronchiseptica* exist as multicellular communities adherent to the nasal epithelium and display distinct three dimensional architecture. Moreover, these biofilms are surrounded by a self-produced extracellular matrix composed of the Bps polysaccharide thereby satisfying the definition of *in vivo* biofilms [9], [10]. While the nose has been clearly established by us to be a site where *Bordetella* form biofilms, it is not known whether these bacteria exist in this form in the trachea. Since FHA promotes efficient attachment of *Bordetella* to the ciliated respiratory epithelia and contributes to the colonization of the lower respiratory tract [31], [49], [50], we investigated its role in the development of multi-cellular biofilm like structure in the nose and trachea. Based on our *in vitro* results, which demonstrate a role for FHA during both attachment and mature biofilm development, we hypothesized that a similar result would be obtained *in vivo*. We therefore examined two time points post-inoculation, an early time point (1 day) to determine the role of FHA in direct attachment to the epithelial surface and a late time point of 7 days. Inoculation with 5×10^5^ CFUs of wild-type strain of *B. pertussis* results in maximal infection of the entire respiratory tract by seven days and we hypothesize this time point to represent a mature biofilm phenotype. At 1 and 7 days post-inoculation, the nasal septum and trachea of mice inoculated with either the WT or the Δ*fhaB* strains were excised and dissected into two equivalent sections. One section was subjected to analysis of colonization profiles by enumerating the CFUs, whereas the other section was examined for the presence of biofilms by probing for *B. pertussis* cells adherent to the respiratory epithelium.

**Figure 6:**
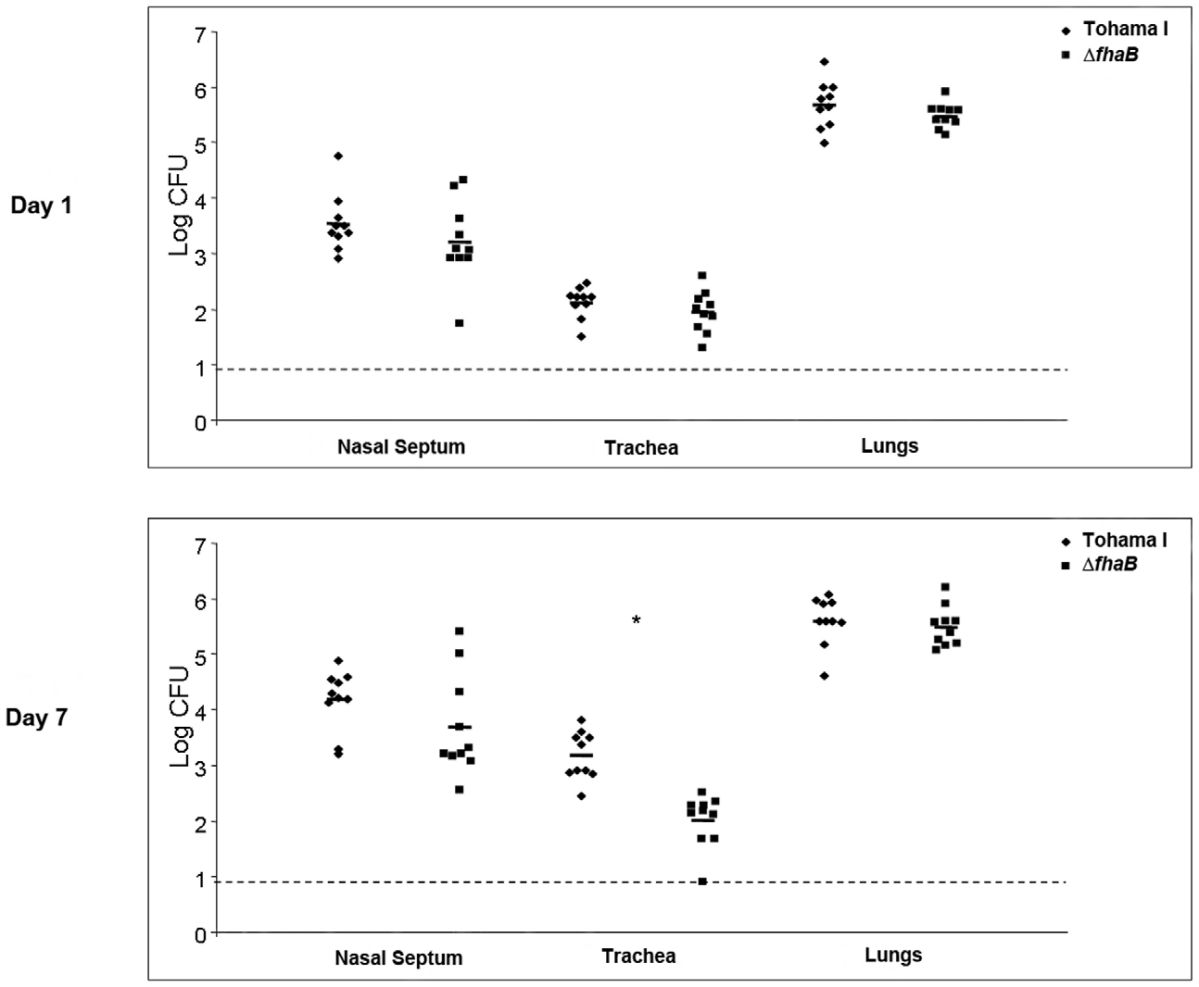
Colonization of the murine respiratory tract by the WT and Δ*fhaB* strains. Groups of five, 6 to 8 week old C57BL/6 mice were intranasally inoculated with containing 5×10^5^ CFUs of either the WT or the Δ*fhaB* strain in 50 µl. After 1 (A) or 7 (B) days, mice were sacrificed and colonization was assessed for the nasal septum, trachea, and lungs. Horizontal bars represent the mean for each group. The dashed lines indicate the limit of detection. The experiment was performed in duplicate with all mice from both experiments being represented. An asterisk indicates significant differences between the WT and the Δ*fhaB* mutant (*P* value <0.05; unpaired two-tailed Student's *t*-test).

At one day post-inoculation, no statistical differences were observed in the numbers of recovered CFUs of either the WT or the Δ*fhaB* strain from the nose or the trachea (Fig. 6). However, compared to the nose, both the strains colonized the trachea in lower numbers. These results are similar to a previous study in which the maximal number of CFUs harvested from the trachea one day post-inoculation with the WT strain of *B. pertussis* was less than 100 [51]. At seven days post-inoculation and consistent with previous results, the mutant strain displayed a significantly reduced ability to colonize the trachea (*P* = 0.00018, Student's *t*-test) [32], [52]. However, although there was an overall reduction in the colonization of the nose by the mutant strain, this difference was not statistically significant (Fig. 6). We also examined the colonization of the lungs by these two strains. Both the WT and the mutant strain colonized the lungs in large numbers and no statistically significant differences in colonization were observed either at 1 or 7 days after inoculation.

**Figure 7:**
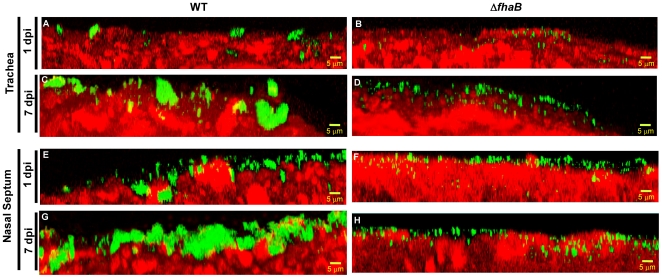
Immunofluorescence microscopy analysis of *B. pert*ussis biofilm formation within the murine respiratory tract. Groups of 6-week-old C57BL/6 mice were intranasally inoculated with 50 µl containing 5×10^5^ CFUs of either the WT or Δ*fhaB* strain grown under Bvg^+^ phase conditions. Sections of trachea and nasal septum were harvested at 1 or 7 days post-infection from infected animals, immediately fixed, and probed with rat anti-*Bordetella* serum followed by a donkey anti-rat secondary antibody conjugated to Alexa Flour 488 (which stains bacteria green). To determine the localization of the host epithelium, specimens were stained for F-actin using phalloidin conjugated to Alexa Fluor 633 (which stains the epithelium red). For visualization with CLSM, those tissues corresponding to infected animals that showed similar levels for colonization by the WT and the Δ*fhaB* mutant were chosen. For each specimen, image stacks were obtained from at least ten areas of the trachea and nasal septum. (A–H) Each micrograph is a Z reconstruction produced from a representative stack of Z-section images.

For examination of biofilms, we chose tissues from infected animals that showed similar levels of colonization by the WT and the mutant strains. In addition to visually examine the samples by CSLM, we also utilized the COMSTAT software to quantitate the differences in biomass and mean thickness between the WT and Δ*fhaB* strains (Table 3). Examination of nasal septum and trachea at 1 day post-inoculation, revealed that the WT cells mainly formed small interspersed clusters on the tracheal and nasal epithelium (Figs. 7A and 7E). The discrepancy between similar numbers of CFUs harvested and the observed biofilm phenotype may be explained by hypothesizing that the WT exists as localized cell clusters which occupy a small portion of the surface, while the Δ*fhaB* strain is primarily single cells covering a larger surface area of the epithelia, thereby resulting in equivalent number of cells being harvested from the two sites. Consistent with this hypothesis, quantitative analyses using COMSTAT did not reveal any statistical differences in the biofilm biomass formed over the two epithelia whereas the mean thickness of the biofilms formed by the WT strain was greater than that of the biofilms formed by the mutant strain (Table 3). Control samples, which consisted of nasal and tracheal tissues from mice infected with PBS, only displayed reactivity to actin, the epithelia marker (data not shown).

**Table 3:** COMSTAT analysis of CSLM images generated from trachea and nasal septum tissues isolated from mice infected with either the WT or Δ*fhaB* strain.

	Parameter^a^	Biomass (µm^3^. µm^−2^)		*P* value^c^	Mean thickness (µm)		*P* value^c^
Tissue	dpi^b^ Strain	WT	Δ*fhaB*		WT	Δ*fhaB*	
**Trachea**	**1**	0.594 (0.189)	0.246 (0.138)	0.0615	2.675 (0.689)	0.910 (0.361)	0.017
	**7**	3.15 (0.851)	0.721 (0.243)	0.0089	4.460 (1.221)	1.864 (0.5509	0.0283
**Nasal Septum**	**1**	1.187 (0.346)	0.837 (0.293)	0.252	3.042 (0.881)	1.531 (0.286)	0.0475
	**7**	4.861 (1.142)	0.802 (0.195)	0.0037	7.233 (1.509)	1.820 (0.364)	0.0079

a Average values of parameters obtained from COMSTAT analysis of at least six CLSM image stacks derived from duplicated experiments. Standard errors are given in parentheses.

b dpi; days post-infection.

c *P* values were determined using an unpaired two-tailed Student's *t*-test.

After 7 days of inoculation, the differences in the spatial arrangement between the Δ*fhaB* and WT cells were found to be markedly more pronounced than those observed one day post-inoculation for both the organs. Consistent with *in vitro* data, the WT strain formed thick, irregularly shaped colonies on focal areas of the apical surfaces of both the nose and the trachea, resembling structures that are characteristics of biofilms (Figs. 7C and 7G). In contrast, the Δ*fhaB* strain formed only minute clusters or thin spikes that did not reach the thickness or the biomass of the biofilms observed in the WT infected animals (Figs. 7D, 7H and Table 3).

We also considered the possibility that the observed biofilm defect of the Δ*fhaB* strain could be the result of poor recognition of this strain by the rat anti-*Bordetella* serum. To address this caveat, we carried out immunofluorescence analyses with *in vitro* grown cultures grown under Bvg^+^ phase conditions. Our results showed that both the WT and the mutant strains were equally fluorescent, leading us to conclude that both strains are recognized with similar efficiency by the rat anti-*Bordetella* serum (data not shown).

These results not only confirm our previous results documenting nasal biofilms of *B. pertussis* [10], but also demonstrate for the first time that this bacterium can form biofilms in the trachea. Moreover, these also provide strong evidence highlighting the critical role of FHA in the formation and maintenance of biofilms in the mouse respiratory tract.

## Discussion

Biofilm formation by the human pathogen *B. pertussis* is a largely unexplored field of research and necessitates detailed studies of factors that regulate this complex developmental program. In the present study, we sought to gain insights into the structural role of surface-associated FHA in biofilm formation. Specifically, we dissected its role in the distinct biofilm developmental stages of *B. pertussis* both *in vitro*, using abiotic substrates, and in the respiratory tract by employing a murine model of respiratory biofilms.

The absence of FHA was found to strongly impair the ability of *B. pertussis* to accumulate biofilm biomass *in vitro*. This was independent of the nature of the surfaces used since this phenotype was observed on both polypropylene and borosilicate surfaces. In various bacterial biofilm developmental processes, the functions of many of the surface proteins such as LapA of *P. fluorescens*, Type 1 fimbriae of *E. coli*, and MshA pili of *V. cholerae* [24], [53], [54] are generally restricted to early biofilm stages, essentially to the steps in which bacteria establish reversible, followed by irreversible, surface attachment. Our studies in which bacterial attachment was separated from post-attachment steps revealed that the impaired biofilm-forming capacity of the Δ*fhaB* mutant was not limited only to the early surface attachment. The lack of FHA altered the formation of cellular aggregates or biofilms. Microscopy studies revealed that in contrast to the robust biofilm architecture of the WT strain that consisted essentially of thick and large microcolonies surrounded by open channels, the biofilms formed by the mutant strain were represented mostly by single cells dispersed over the surface or by very small cell clusters at late time points. In addition, our results showed that Δ*fhaB* cells were unable to be integrated with WT cells into the community, demonstrating that FHA on the surface of the WT bacterium was unable to compensate efficiently for surface deficiency of FHA on the Δ*fhaB* bacterium. Similar results were obtained for *Salmonella enterica* serovar Enteritidis, for which the presence of the BapA adhesin on the cell surface is required to mediate the incorporation of bacteria into the biofilm microcolonies [55]. In line with a major role for surface-associated FHA in formation of *Bordetella* biofilms, antibody-mediated blocking of FHA present on either the planktonic or the biofilm bacteria abrogated the integration of bacterial cells into the biofilms. We conclude that FHA plays a crucial role in initial attachment and subsequent development of highly organized mature biofilms by governing both cell-substrate and cell-cell interactions.

Surprisingly, we found that free FHA reduced the attachment of WT bacteria to the biofilms, but did not further reduce the low residual attachment of the Δ*fhaB* bacteria. In addition to being surface-associated, FHA is also secreted in the extracellular milieu [30], [39]. Naturally secreted FHA might, through inhibition of bacterial attachment to the biofilms via interaction with FHA or other bacterial factors, play a regulatory role in *Bordetella* biofilm formation.

Recently, Lasa proposed a group of surface proteins, which share several structural and functional features, as an important element of biofilm formation by a number bacterial species [56]. The first member of this group was identified in a *S. aureus* mastitis isolate and was named Bap (Biofilm associated protein) [57]. The authors described common features for all Bap-related proteins: i) they are present on the bacterial surface; ii) they are of high molecular weight; iii) they contain a core domain of tandem repeats; iv) they play a relevant role during bacterial infectious processes and v) they confer on bacteria the capacity to form a biofilm. Whereas evidence from a vast number of previous studies indicates FHA fulfils the first four features [31], [42], [58], [59], the results of this study show that it also satisfies the last one. Thus, the demonstrated involvement of FHA at different developmental events of *B. pertussis* biofilm formation emerges as an important aspect of this virulence factor. Remarkably, FHA of *Bordetella* does not appear to be an isolated case. The recent finding that other proteins sharing common features with FHA (i.e., CdrA in *P. aeruginosa*, and XacFhaB in *Xanthomonas axonopodis* pv. Citri) are involved in biofilm formation [40], [41], suggests that an increasing number of proteins in other bacteria which are non-homologous in primary amino acid sequence may satisfy many of the structural and functional attributes of Bap-like proteins.

Despite a large body of information available on the contribution of surface proteins to biofilm growth on different artificial surfaces *in vitro*, little is known about their function in formation of biofilms in host organs. The product of the *pilA* gene of *Haemophilus influenzae* was shown to function in forming stable biofilms in the chinchilla middle ear [60]. Because the *pilA* mutant led to unstable biomass in the chinchilla ear, it was not possible to determine the precise mechanistic role of PilA in organ biofilms. In *B. bronchiseptica*, FHA was found to promote colonization and microcolony formation on the nonciliated olfactory epithelium [36]. Adherence of *Bordetella* to cilia is a critical step in the initiation of a non-invasive mucosal infection. In *in vitro* organ cultures, experimental infections of animals and in human patients, both *B. bronchiseptica* and *B. pertussis* preferentially and sometimes exclusively attach to the ciliated respiratory epithelium [19], [61], [62]. In this study, we show that *B. pertussis* FHA plays an important role in biofilm growth on ciliated nasal epithelium and trachea. Previously, we have shown that the observed biofilm-like structures of *B. pertussis* are characterized by the production and co-localization of the Bps polysaccharide, a component of the biofilm matrix [10]. The present results combined with our previously published data thus strongly suggest that in addition to the previously demonstrated role of FHA in promoting adherence to respiratory tissues [31], [63], [64], it also plays a role in ensuring the long-term tissue adherence by formation of surface adherent biofilm communities in the respiratory tract. This may allow *Bordetella* to escape immune defenses operative in the nose and the trachea.

Although initially described as an adhesin, FHA has now emerged as a multipurpose virulence factor capable of mediating *B. pertussis* invasion of host cells and as an immunomodulatory molecule [33], [34], [35]. The current results documenting the contribution of FHA in the initiation and maturation of *B. pertussis* biofilms represent a unique example of the intricate and versatile role played by a single bacterial virulence factor in the emergence of a highly successful respiratory pathogen.

## Materials and Methods

### Bacterial strains and growth conditions

*B. pertussis* B213, a streptomycin-resistant (Sm^r^) derivative strain of Tohama I (WT) [65]; BPSM, a streptomycin- and nalidixic acid-resistant (Sm^r^; Nal^r^) strain derived from Tohama I (WT) [66], and BPGR4, a BPSM derivative mutant strain lacking expression of FHA (Δ*fhaB*) [43], were used in this study. The Δ*fhaB* strain used for mouse infection studies was also derived from Tohama I and has been described previously [67]. The strains were cultured and maintained on Bordet-Gengou agar (BGA) (Difco Laboratories, Detroit, MI) supplemented with 15% (v v^−1^) defibrinated sheep blood. For both planktonic and biofilm growth conditions, bacteria were grown in Stainer-Scholte (SS) broth and under Bvg^+^ phase conditions. When appropriate, antibiotics were added to the media at the followings concentrations: streptomycin (Sm), 100 µg ml^−1^; nalidixic acid (Nal), 40 µg ml^−1^; gentamycin (Gm), 10 µg ml^−1^.

Planktonic cultures were performed by inoculating bacteria into Erlenmeyer flasks containing SS broth, adjusting the optical density at 650 nm (OD_650_) to 0.15 and incubating the flaks at 37°C overnight under shaking conditions (160 rpm).

### Green fluorescent protein (GFP) labelling of bacteria

*B. pertussis* B213, BPSM and BPGR4 strains were transformed with plasmid pCW504, herein referred to as pGFP, which directs expression of GFP from a constitutive *B. pertussis* promoter [46]. Plasmid pGFP was mobilized into various *B. pertussis* strains by triparental mating using pRK2013 as a helper plasmid. Ex-conjugates were selected on BGA plates containing Gm and Sm or Nal. Randomly selected colonies containing pGFP were grown in SS broth with Gm and were analyzed for GFP expression using fluorescence. One of the GFP-expressing clones corresponding to each of the strains was chosen for experimental analysis. A plasmid stability test revealed that pGFP was stable over 60 generations of growth under nonselective pressure.

### Attachment and biofilm growth conditions

For quantitative analysis of attachment and biofilm growth of *B. pertussis* strains, we utilized glass column systems (φ = 3 cm, W = 18 cm) packed with polypropylene beads (6 g; φ = 4.2 mm; H = 2 mm; Petroken, Argentina), which constituted the growth support. The culture procedure was performed as indicated previously [11]. Briefly, for each strain or culture condition, four column systems were inoculated with a 10-mL planktonic cell suspension at an OD_650_ of 1.0 (1×10^9^ CFUs ml^−1^). Alternatively, when the number of attached cells for the WT strain was required to equal the population of initial adherent Δ*fhaB* mutant strain, the size of the WT inoculum was reduced from 1×10^9^ to 4×10^8^ CFUs ml^−1^. This inoculum size was determined to result in a population of initial adherent WT strain similar to that exhibited by Δ*fhaB* strain with a high inoculum (1×10^9^CFUs ml^−1^). Bacterial cells were allowed to attach (static incubation) to the beads for 4 h at 37°C. After this, beads from two columns per strain or culture condition were collected and analysed for bacterial attachment quantifying the adhered biomass by crystal violet (CV) staining. Cell suspensions of the other two remaining columns per strain or culture condition were drained to remove non-adhered cells and replaced by 10 ml of fresh SS broth. Columns were then incubated aerobically by supplying air at an appropriate flow rate during 72 h or 96 h (when indicated) at 37°C. The growth media were replaced by fresh ones every 24 h. After 72 or 96 h (when indicated) of cultivation, the two columns per strain or culture condition were analysed for biofilm formation by collecting the beads and quantifying the adhered biomass by CV staining or viable cell counting.

For viable cell count determination, the adhered cells were gently washed and detached from beads by slight agitation in PBS buffer. Serial dilutions of cell suspensions were then plated on BGA plates. The number of colony forming units per unit area (CFU cm^−2^) was calculated considering dilutions, surface area of the bead and number of beads.

Attachment of WT and Δ*fhaB* strains to glass surface was assayed in petri dishes containing glass coverslips. For each *B. pertussis* strain, a 15-ml suspension of GFP-expressing cells [pGFP] at an OD_650_ of 1.0 (1×10^9^ CFUs ml^−1^) was inoculated into a petri dish containing 3 coverslips. Bacteria were allowed to attach to the surface under static conditions for 4 h at 37°C. Then, each coverslip was carefully washed to removed non-adhered cells, fixed with 2% (v v^−1^) glutaraldehyde, and visualized by fluorescence microscopy.

Biofilm development by WT and Δ*fhaB* strains was also assayed by using a continuous-flow culture system, which consisted of a chamber (L = 75 mm; W = 25 mm; H = 3 mm) having two glass surfaces, one being a microscope slide and the other being a glass coverslip serving as the substratum. Each system was sterilized in autoclave and dried under a flow of sterilized air. The culture procedure was performed as indicated previously [12]. Briefly, for each strain, at least two continuous-flow chambers were inoculated with 5-ml suspensions of GFP-expressing cells [pGFP] at an OD_650_ of 1.0 (1×10^9^ CFUs ml^−1^). Bacteria were allowed to attach for 4 h at 37°C prior to initiating the flow. The sterile SS medium, which was kept stirred and aerated at 37°C, was pumped through the flow chambers with a peristaltic pump at a flow rate of 0.1 ml min^−1^. Biofilms were visualized *in situ* by fluorescence microscopy every 24 h for 3 days. Alternatively, to investigate architectural aspects, biofilms were also examined by Confocal Laser Scanning Microscopy (CLSM).

### Co-inoculation experiments

For simultaneous co-inoculation experiments, 5-ml planktonic cell suspensions at an OD_650_ of 1.0 (1×10^9^ CFUs ml^−1^) corresponding to the B213[pGFP] (WT, Gm^r^) strain were mixed with similar suspensions of the BPGR4 (Δ*fhaB*, Nal^r^) or the BPSM (WT, Nal^r^) strains, and then introduced into independent glass column systems packed with polypropylene beads. The biofilm experiments were conducted as described above. After 72 h of cultivation, the number of cells corresponding to each strain was determined by plating aliquots of appropriate dilutions of the whole cell population recovered from the polypropylene beads on selective BGA plates containing Gm for B213[pGFP] (WT) and Nal for BPGR4 (Δ*fhaB*) or BPSM (WT).

In sequential co-inoculation experiments, 10-ml planktonic cell suspensions at an OD_650_ of 1.0 corresponding to the B213[pGFP] (WT) strain were inoculated into two glass column systems and allowed to establish biofilms on the polypropylene surface for 24 h. After this, 10-ml planktonic suspensions (OD_650_ = 1.0) of the BPGR4 (Δ*fhaB*) or BPSM (WT) strains were independently inoculated into each system, allowed to attach for 4 h, and then drained to remove non-adhered cells and replaced by fresh SS broth. After that, the biofilm experiments were conducted as described above determining at the 72-h time point the number of sessile cells corresponding to each strain. Data are presented as fractions of the total bacteria recovered.

### Attachment to pre-formed biofilms

Round glass coverslips (diameter: 12 mm; thickness: 0.13 mm) were introduced in a 20-ml continuous-flow chamber system. The system was sterilized in autoclave and dried under a flow of sterilized air. The biofilm culture chamber was inoculated with a suspension of BPSM (WT) cells at an OD_650_ of 1.0 (1×10^9^ CFUs ml^−1^). Bacteria were allowed to attach for 4 h prior to initiating the flow (it removed planktonic bacteria leaving those attached to the surface). Sessile cells were allowed to form biofilm for 24 h. Coverslips containing the attached biofilms were removed from the chamber, rinsed with SS medium, and placed into individual wells of 24-well microtiter plates. Where indicated, coverslips were blocked with 1% bovine serum albumin (BSA) (Sigma, St. Louis, Mo.), washed with SS, and then incubated with dilutions of an anti-FHA serum (1∶1000; 1∶500, 1∶250) (product code: 97/564; National Institute for Biological Standards and Control, UK) for 1 h at 37°C. Next, 500-µl planktonic cell suspensions (1×10^7^ CFUs ml^−1^) of BPSM[pGFP] (WT) or BPGR4[pGFP] (Δ*fhaB*) strains, with or without pre-incubation for 1 h at 37°C with either dilutions of the anti-FHA serum (1∶1000; 1∶500, 1∶250) or purified FHA (product code: 90/520, purity: >98%; NIBSC, UK) (final concentration of 5, 10 and 20 µg ml^−1^), were added into individual wells of the plate containing the coverslips. After each pre-incubation step, with the respective sera or purified FHA, the bacteria or the biofilms were washed with SS. GFP-tagged bacteria in the planktonic suspension were allowed to attach to microcolonies on the coverslips for 4 h at 37°C. Then, non-attached bacteria were removed by rinsing the coverslips with SS. The samples on the coverslips were fixed with 2% (v v^−1^) glutaraldehyde. To visualize the GFP-expressing planktonic bacteria that remained attached to the biofilms, multi-channel (transmitted light and fluorescence) CLSM images were acquired. The attachment level was determined by enumerating those GFP-tagged bacteria that colocalize with the biofilms through the analysis of the multichannel images using ImageJ (U.S. National Institutes of Health, Bethesda, MD, USA) in conjunction with the ITCN plug-in and COMSTAT (Heydorn et al., 2000). At least six randomly selected microscopic fields per coverslips and four coverslips from four independent experiments were analysed.

### Quantification of bacterial attachment and biofilm formation

*B. pertussis* cell biomass adhered to polypropylene bead surface was quantified by crystal violet (CV) staining. The protocol was adapted from that described by O'Toole and Kolter [68]. When early attachment and biofilm growth were investigated, sessile biomass was quantified at 4, 72 or 96 h (when indicated) of incubation respectively. Briefly, beads with adhered biomass were gently rinsed twice with PBS to remove non-attached cells, air dried, and then carefully transferred to glass tubes. Cells were stained by addition of 4.5 ml of CV 0.1% (w v^−1^) for 15 min. The stain was removed by exhaustive washing with distilled water. Then, 9 ml of decolouring solution of ethanol/acetone (80∶20) was added to each tube. The absorbance of the eluted stain was measured at 590 nm (A_590_).

### Fluorescence microscopy

Biofilms developed on the base of the flow chambers were visualized *in situ* by fluorescence microscopy. Visualization was conducted at a magnification of 400× or 1000× by using a Leica DMLB microscopy (Leica Microsystems, Wetzlar, Germany) equipped with a standard Blue/Green/Red filters set (excitation: 400/20, 495/15, 570/50 nm) and with a charge coupled device (CCD) digital camera. For capturing and displaying of images, the Leica IM50 software was utilized.

### Confocal laser scanning microscopy (CLSM) and image analysis

To study architectural features of biofilms developed *in vitro* on the base of the flow chambers, on coverslips, or formed *in vivo* on murine nasal and tracheal tissues, a Zeiss LSM510-Axiovert 100 M confocal laser scanning microscope (Carl Zeiss, Germany) was used. Stacks of Z-section images were viewed and processed using the Carl Zeiss LSM5 Image Browser version 3.2.0 and/or the Imaris Software. Images of *in vitro* biofilms were acquired from *B. pertussis* cells expressing GFP [pGFP]. The detection of the emitted light was performed by sequentially scanning with settings optimal for GFP (488-nm excitation with argon laser line and 505-nm long-pass emission). In each experiment, images were acquired from random points by using a plan-neofluar ×100/1.3 oil immersion objective, at 1 µm z-intervals down through the biofilm. In order to acquire quantitative information of the mature biofilm structure, CLSM images were analyzed by the computer program COMSTAT [45]. To obtain statistically representative results, triplicate experiments of 72 h-old biofilms were performed.

To visualize *in vivo B. pertussis* biofilms, bacteria were probed with rat anti-*Bordetella* serum followed by a secondary anti-rat antibody conjugated to Alexa Fluor 488, whereas tracheal and nasal septum tissues were stained for F-actin using phalloidin conjugated to Alexa Fluor 633 [9]. The tissues were then mounted in ProLong 9 Gold anti-fade reagent (Invitrogen, Carlsbad, CA, USA) in four-chambered cover glass. The detection of the emitted light was performed by sequentially scanning with settings optimal for both fluorophores. Images were acquired from at least 10 areas of each tissue section by using a C-Apochromat 63×/1.2W objective, at 1 µm z-intervals down through the section of the tissue. Stacks of Z-section images were viewed and processed to create Z reconstructions using the Carl Zeiss LSM5 Image Browser version 3.2.0. In order to acquire quantitative information of *in vivo B. pertussis* biofilms, CLSM images were also analyzed by the computer program COMSTAT [45].

### Animal experiments

Groups of five 6-week-old female C57BL/6 mice (Jackson Laboratory) were lightly sedated with isoflurane (Butler) and were intranasally inoculated with either 50 µl of sterile PBS alone or with 5×0^5^ CFUs of Tohama I or the isogenic Δ*fhaB* strain. As stated above, for animal experiments, the strains were grown under Bvg^+^ phase conditions. Immediately after inoculation of the animals, an aliquot was plated to ensure that the strains were in the Bvg^+^ phase. At designated times post-inoculation, mice were euthanized, and the nasal septum, trachea and the lungs were excised. The nasal septum and the trachea were transversally cut into two equivalent sections. One of the sections of these two tissues was fixed in 10% normal buffered formalin, and processed for microscopy as described below. The remaining section of the nasal septum and the trachea as well as the lungs were homogenized in PBS and plated onto BGA plates containing Sm (50 µg ml^−1^). Colonies were enumerated after 4 days of growth at 37°C. All animal experiments were carried out in accordance with institutional guidelines and were repeated in duplicate. Statistical analysis was carried using an unpaired two-tailed Student *t* test.

### Immunofluorescent labelling of *B. pertussis* biofilms formed *in vivo*

Immunofluorescent experiments were carried out as previously described [9], [10]. Briefly, after fixation, one section of nasal septum and the trachea were washed with PBS. At this step the tracheal section was carefully split open longitudinally. After this, the sections of both tissues were blocked with 5% normal donkey serum for 30 min. The tissues were then incubated with rat anti-*Bordetella* serum (1∶1,000) for 2 h at room temperature. This serum was collected from a rat 30 days after inoculation with a Bvg^+^ phase-locked derivative of RB50. After this, the tissues were washed five times with PBS, and subsequently incubated for 2 h at room temperature with a donkey anti-rat secondary antibody conjugated to Alexa Fluor 488 (1∶200). Samples were again washed five times and fixed for 30 min in 10% normal buffered formalin to prevent antibody-antigen dissociation during microscopy. Tissues were washed with PBS, permeabilized with 0.1% Triton X-100, and stained for eukaryotic F-actin with a 1∶40 dilution of phalloidin conjugated to Alexa Fluor 633 for 30 min. Samples were visualized by CLSM as described above. For visualization, those tissue sections corresponding to infected animals that showed similar levels for colonization by the WT and the Δ*fhaB* mutant were chosen.

### Ethics statement

Animal husbandry and experimental procedures were performed in accordance with Public Health Service policy and the recommendations of the Association for Assessment and Accreditation of Laboratory Animal Care and approved by the Wake Forest University Health Sciences Institutional Animal Care and Use Committee.

## Supporting Information

Figure S1 **Time course of the growth of the** ***B. pertussis*** **Tohama I derivatives BPSM and B213 in shaking cultures.** Shaking cultures were initiated by inoculating bacteria into 2 L-Erlenmeyers flasks containing 300 ml of SS broth, adjusting the optical density at 650 nm (OD_650_) to 0.1. The flasks were incubated at 37°C under shaking conditions (160 rpm). One-milliliter aliquots of cell suspensions were taken every 2 h for OD measurements.
(TIF)

Figure S2 **Biofilm formation by** ***B. pertussis*** **Tohama I derivatives BPSM and B213 on polypropylene.** Biofilm cultures were performed in glass column systems containing polypropylene beads. (A) Image of CV-stained cells of BPSM and B213 strains adhered to polypropylene beads after 72 h of cultivation in glass column systems. (B) Biofilm biomass accumulated by each strain over the polypropylene beads after 72 h of cultivation was stained with CV 0.1% (v v^−1^). The CV stain associated with cells was solubilized in ethanol/acetone (80∶20) and the resulting solution was subjected to measurement of the absorbance at 590 nm. The data are the means ± standard deviations of three independent experiments.
(TIF)

Figure S3 **Biofilm formation by B213[pGFP] and B213** ***B. pertussis*** **strains on polypropylene.** Biofilm cultures were performed in glass column systems containing polypropylene beads. (A) Images of polypropylene beads containing adhered cells of B213[pGFP] and B213 strains which were stained with crystal violet (Upper panel) or exposed to UV light (Lower panel). (B) Biofilm biomass accumulated by each strain over the polypropylene beads after 72 h of cultivation was stained with CV 0.1% (v v^−1^). The CV stain associated with cells was solubilized in ethanol/acetone (80∶20) and the resulting solution was subjected to measurement of the absorbance at 590 nm. The data are the means ± standard deviations of three independent experiments.
(TIF)

Figure S4 **Association of** ***B. pertussis*** **planktonic cells with the biofilms after pre-incubated with normal sheep serum.** (A) GFP-tagged planktonic bacteria of the WT (BPSM) strain, pre-incubated with a 1∶250 dilution of normal sheep serum, followed by incubation for 4 h with 24-h-old WT biofilms. (B) 24-h-old WT biofilms were pre-incubated with a 1∶250 dilution of normal sheep serum, and then subjected to a 4-h incubation period with planktonic GFP-tagged WT bacteria. GFP-expressing bacteria attached to the biofilms were visualized by acquiring multi-channel (transmitted light and fluorescence) CLSM images. At least six microscopic fields per coverslips and four coverslips per condition were analysed. A representative merged image for each condition is shown.
(TIF)
