# Supplementary figures and images for: FHA-Mediated Cell-Substrate and Cell-Cell Adhesions Are Critical for Bordetella pertussis Biofilm Formation on Abiotic Surfaces and in the Mouse Nose and the Trachea
Source: PLoS One. 2011 Dec 22;6(12):e28811. doi: 10.1371/journal.pone.0028811 (PMC3245231; doi:10.1371/journal.pone.0028811)

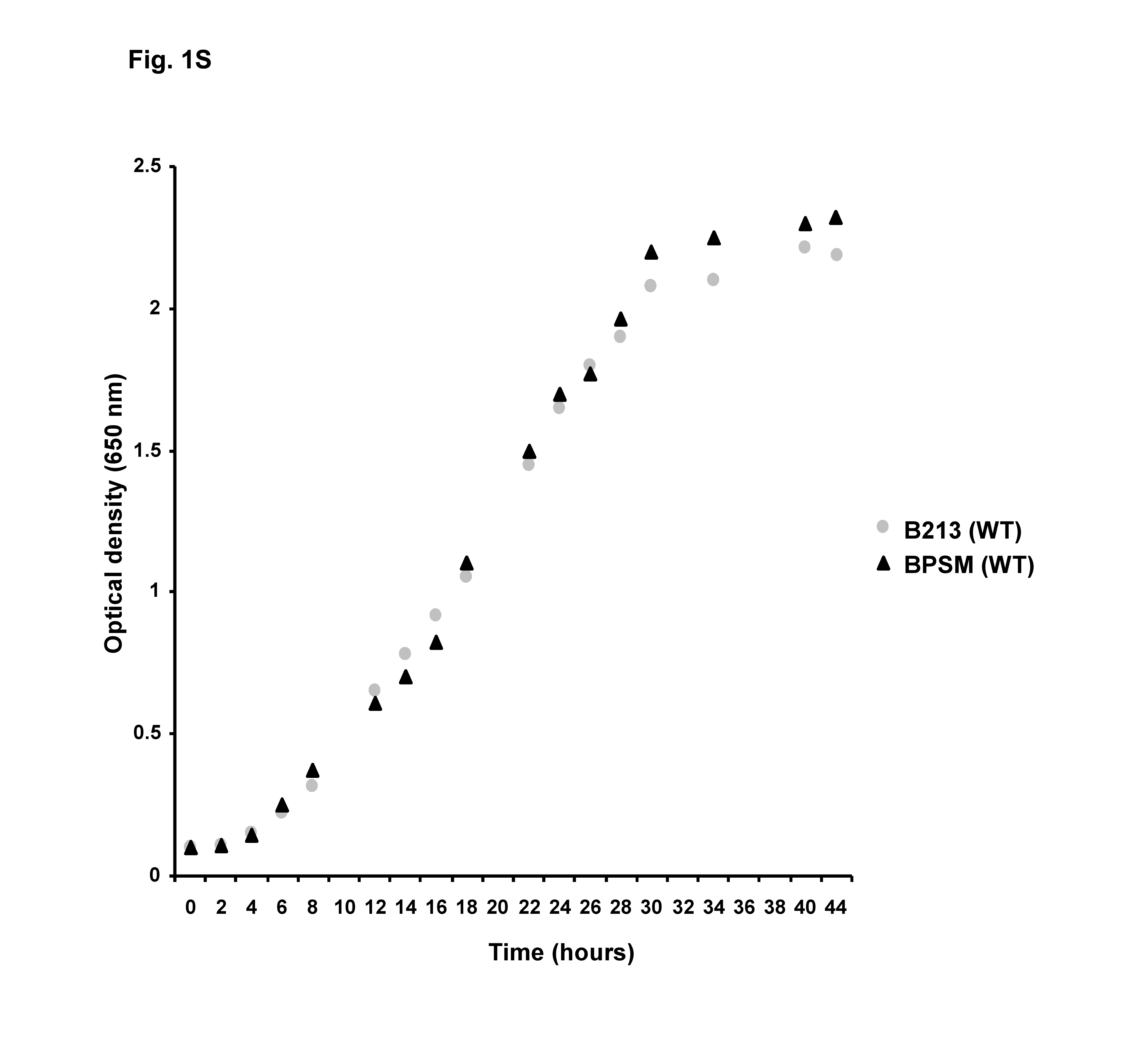

Supplement: Figure S1 — Time course of the growth of the B. pertussis Tohama I derivatives BPSM and B213 in shaking cultures. Shaking cultures were initiated by inoculating bacteria into 2 L-Erlenmeyers flasks containing 300 ml of SS broth, adjusting the optical density at 650 nm (OD650) to 0.1. The flasks were incubated at 37°C under shaking conditions (160 rpm). One-milliliter aliquots of cell suspensions were taken every 2 h for OD measurements. (TIF) [file pone.0028811.s001.tif]

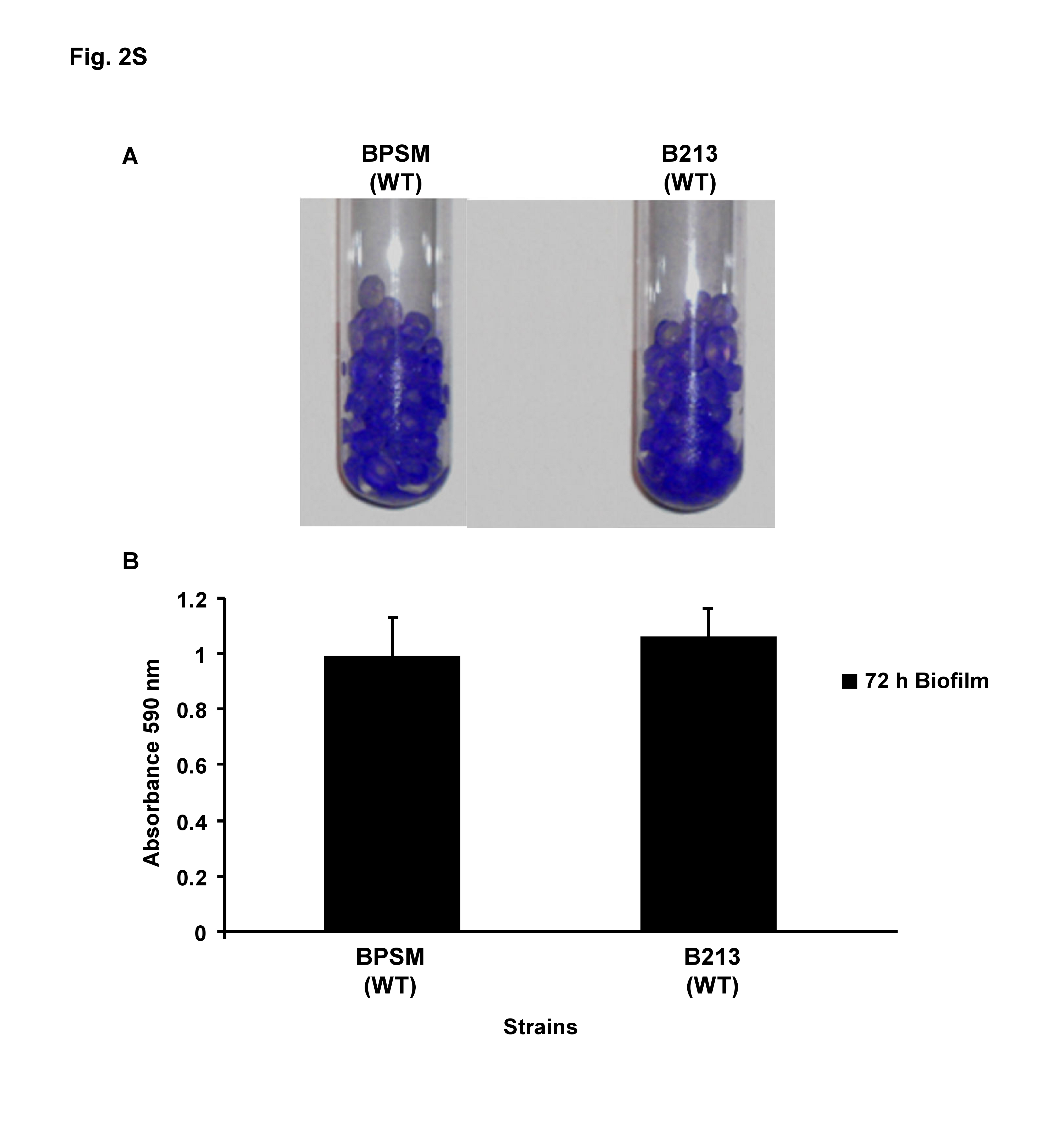

Supplement: Figure S2 — Biofilm formation by B. pertussis Tohama I derivatives BPSM and B213 on polypropylene. Biofilm cultures were performed in glass column systems containing polypropylene beads. (A) Image of CV-stained cells of BPSM and B213 strains adhered to polypropylene beads after 72 h of cultivation in glass column systems. (B) Biofilm biomass accumulated by each strain over the polypropylene beads after 72 h of cultivation was stained with CV 0.1% (v v−1). The CV stain associated with cells was solubilized in ethanol/acetone (80∶20) and the resulting solution was subjected to measurement of the absorbance at 590 nm. The data are the means ± standard deviations of three independent experiments. (TIF) [file pone.0028811.s002.tif]

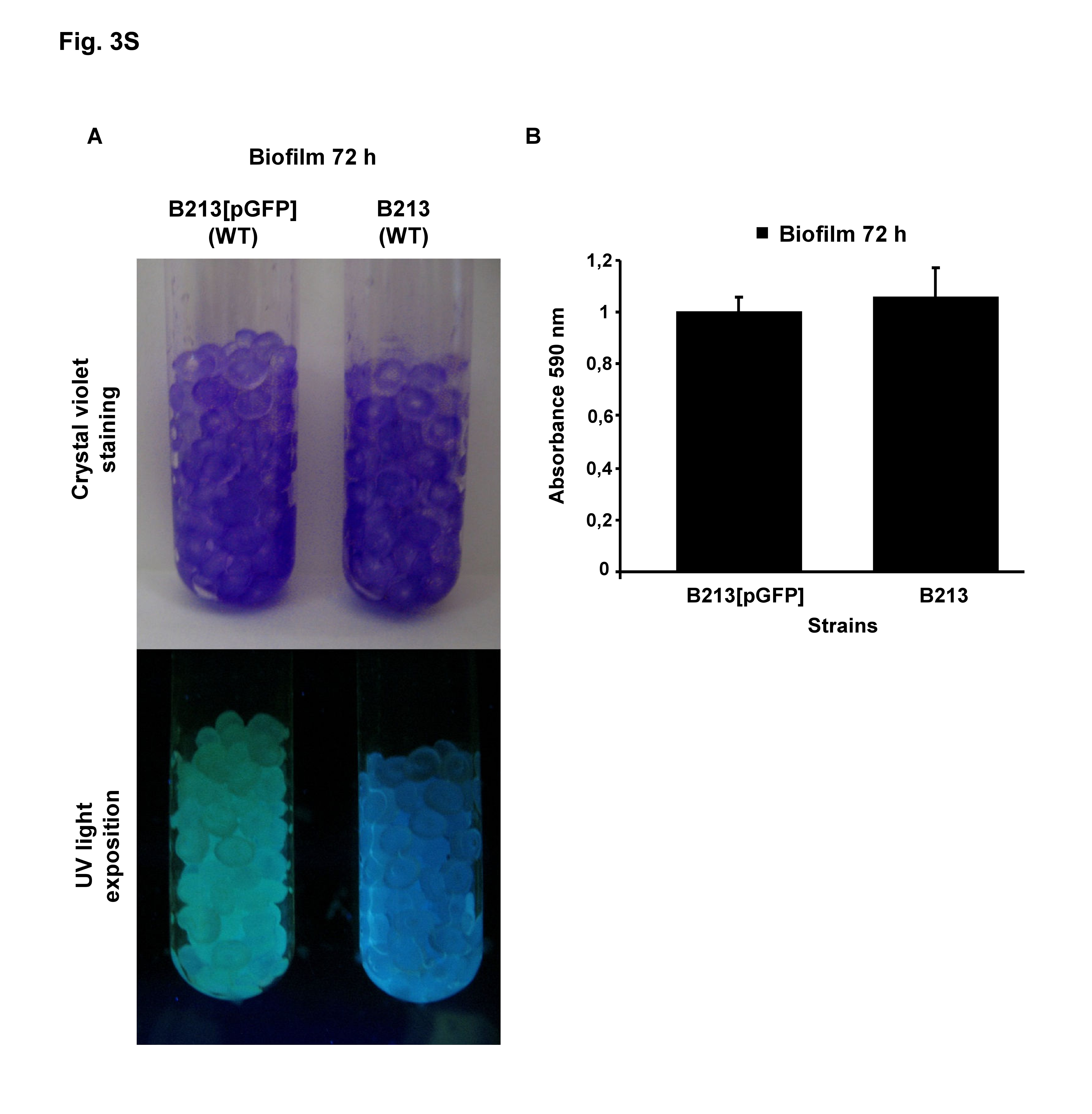

Supplement: Figure S3 — Biofilm formation by B213[pGFP] and B213 B. pertussis strains on polypropylene. Biofilm cultures were performed in glass column systems containing polypropylene beads. (A) Images of polypropylene beads containing adhered cells of B213[pGFP] and B213 strains which were stained with crystal violet (Upper panel) or exposed to UV light (Lower panel). (B) Biofilm biomass accumulated by each strain over the polypropylene beads after 72 h of cultivation was stained with CV 0.1% (v v−1). The CV stain associated with cells was solubilized in ethanol/acetone (80∶20) and the resulting solution was subjected to measurement of the absorbance at 590 nm. The data are the means ± standard deviations of three independent experiments. (TIF) [file pone.0028811.s003.tif]

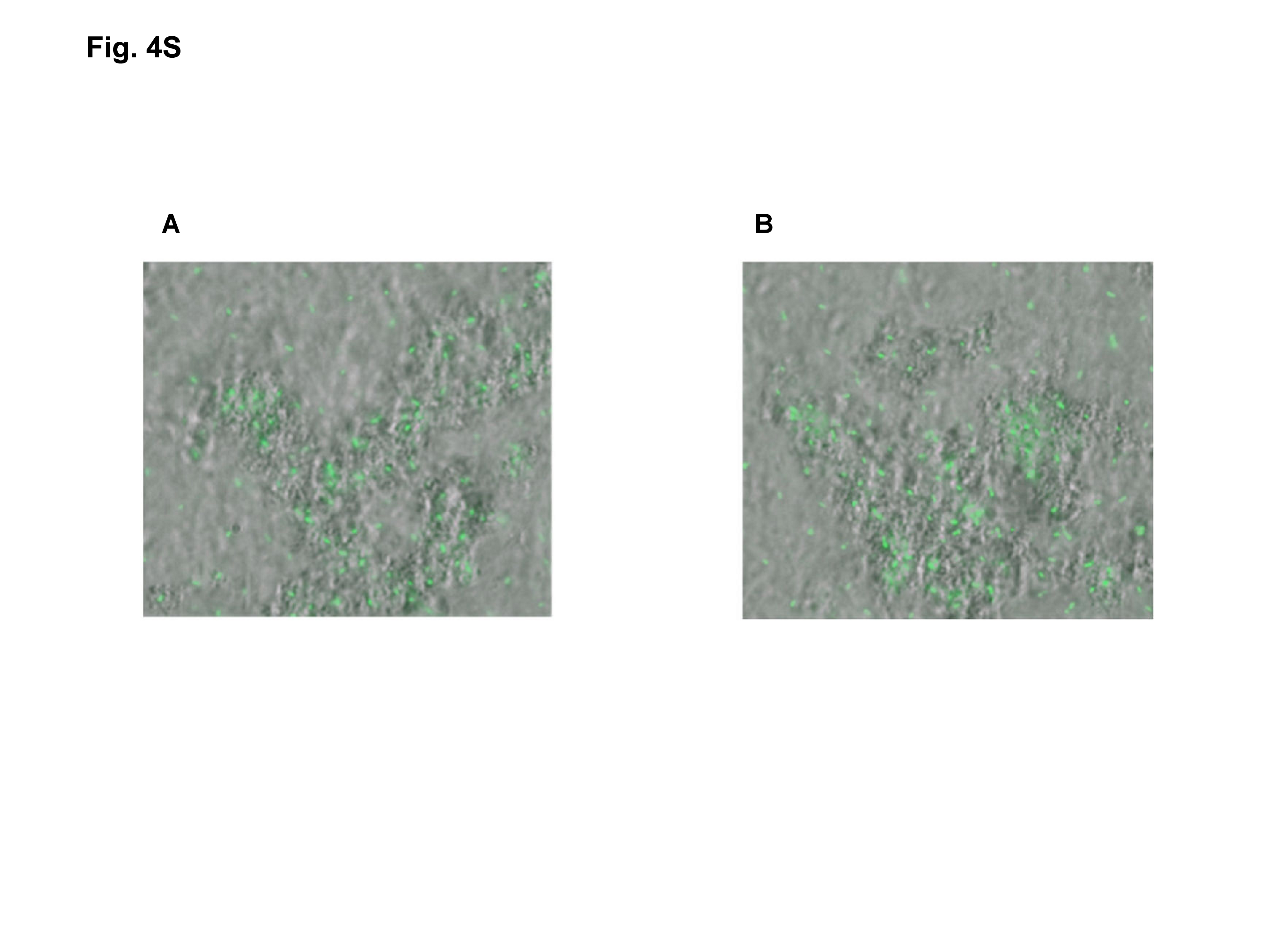

Supplement: Figure S4 — Association of B. pertussis planktonic cells with the biofilms after pre-incubated with normal sheep serum. (A) GFP-tagged planktonic bacteria of the WT (BPSM) strain, pre-incubated with a 1∶250 dilution of normal sheep serum, followed by incubation for 4 h with 24-h-old WT biofilms. (B) 24-h-old WT biofilms were pre-incubated with a 1∶250 dilution of normal sheep serum, and then subjected to a 4-h incubation period with planktonic GFP-tagged WT bacteria. GFP-expressing bacteria attached to the biofilms were visualized by acquiring multi-channel (transmitted light and fluorescence) CLSM images. At least six microscopic fields per coverslips and four coverslips per condition were analysed. A representative merged image for each condition is shown. (TIF) [file pone.0028811.s004.tif]
